# Methane utilization in *Methylomicrobium alcaliphilum* 20Z^R^: a systems approach

**DOI:** 10.1038/s41598-018-20574-z

**Published:** 2018-02-06

**Authors:** Ilya R. Akberdin, Merlin Thompson, Richard Hamilton, Nalini Desai, Danny Alexander, Calvin A. Henard, Michael T. Guarnieri, Marina G. Kalyuzhnaya

**Affiliations:** 1 Biology Department and Viral Information Institute, San Diego State University, San Diego, USA; 2 Institute of Cytology and Genetics and Novosibirsk State University, Novosibirsk, Russia; 3 Metabolon, Inc. 617 Davis Drive, Suite 400, Durham, NC 27713 USA; 4 National Bioenergy Center, National Renewable Energy Laboratory, 15013 Denver West Parkway, MS 3323, Golden, CO 80401 United States

## Abstract

Biological methane utilization, one of the main sinks of the greenhouse gas in nature, represents an attractive platform for production of fuels and value-added chemicals. Despite the progress made in our understanding of the individual parts of methane utilization, our knowledge of how the whole-cell metabolic network is organized and coordinated is limited. Attractive growth and methane-conversion rates, a complete and expert-annotated genome sequence, as well as large enzymatic, ^13^C-labeling, and transcriptomic datasets make *Methylomicrobium alcaliphilum* 20Z^R^ an exceptional model system for investigating methane utilization networks. Here we present a comprehensive metabolic framework of methane and methanol utilization in *M. alcaliphilum* 20Z^R^. A set of novel metabolic reactions governing carbon distribution across central pathways in methanotrophic bacteria was predicted by *in-silico* simulations and confirmed by global non-targeted metabolomics and enzymatic evidences. Our data highlight the importance of substitution of ATP-linked steps with PPi-dependent reactions and support the presence of a carbon shunt from acetyl-CoA to the pentose-phosphate pathway and highly branched TCA cycle. The diverged TCA reactions promote balance between anabolic reactions and redox demands. The computational framework of C_1_-metabolism in methanotrophic bacteria can represent an efficient tool for metabolic engineering or ecosystem modeling.

## Introduction

Climate change concerns linked to increasing concentrations of anthropogenic methane have spiked interest in biological methane utilization as a novel platform for improving ecological and human well-being^1–4^. Methane-utilizing microbes (i.e., methanotrophs) are becoming an attractive and efficient microbial platform for organic manufacturing of renewable fuels and chemicals^3–6^. Recent application of modern metabolic engineering tools has furthered methane-based biotechnology, leading to construction of novel biocatalyst traits with great biotechnological potential^7–9^. The recent progress in this area has also highlighted the need for a deeper understanding of the methane-conversion network organization.

Our knowledge of aerobic methane utilization is founded on a number of remarkable discoveries in the biochemistry, physiology, genetics, and ecology of this unique biological function in Proteobacteria^10–12^. The established network of methane utilization has been revisited through the discovery of high redundancy of core functional oxidation machinery and the incredible flexibility of the redox balancing pathways^13–20^. Access to complete genome sequences of methanotrophic bacteria^21,22^ enables whole-genome metabolic reconstruction and flux-balance modeling^23–25^. Several limitations of the top-down modeling of methylotrophy have been highlighted^24^, including limited knowledge of redox transfer reactions, flexibility of the primary methane oxidation pathways, and lack of data on energy maintenance. Herein, we applied a set of systems-level approaches for comprehensive investigation of the methane utilization network in *Methylomicrobium alcaliphilum* 20Z^R^ (a rifampicin-resistant derivative of the strain 20Z^14–16^). Complete and expert-annotated genomic information, a large set of genetic tools, wide-ranging enzymatic studies, and the availability of whole-genome transcriptomic data make 20Z^R^ an ideal model system for understanding the central metabolic pathways associated with methane oxidation^16,26–34^. *M. alcaliphilum* 20Z^R^ possesses only particulate methane monooxygenase (pMMO) for the first step of methane conversion. The enzyme is a mixed function oxidase, in which one atom from O_2_ goes to methanol and the other to water, requiring the input of 2 electrons and 2 protons. To date the source of electrons is still unknown. The second step of the methane oxidation pathway is catalyzed by a periplasmic pyrroloquinoline quinone-linked methanol dehydrogenase. Two enzymes are predicted, a Ca-dependent MxaFI system and La-dependent XoxF^15^. Genome annotation for the strain 20Z predicts multiple pathways for formaldehyde oxidation, including tetrahydromethanopterin- and tetrahydrofolate-linked pathways, formaldehyde dehydrogenase, and the oxidative ribulose monophosphate cycle^15^. The genome has two clusters encoding NAD-dependent formate dehydrogenases: 1) NAD-linked, tungsten (W)-dependent formate dehydrogenase; and 2) a partial NAD-linked, molybdenum-dependent formate dehydrogenase. Only the first system is expressed under methane-growth conditions, even if W is not supplied. Since formate is typically accumulates in the growth media^16,33^, it could be suggested, that the enzyme is only partially active. The impact of tungsten addition on growth of *Methylomicrobium alcaliphilum* 20Z^R^ has never been evaluated. Genome annotation indicates that metabolic pathways for carbon assimilation are highly branched and include the assimilatory ribulose monophosphate pathway (RuMP) and the partial serine cycle^16^. Two variants of the RuMP have been described in 20Z^16^. Furthermore, it has been demonstrated that 75% of intercellular pyruvate comes from the pyrophosphate (PPi)-mediated Embden-Meyerhof-Parnas pathway (EMP); and 25% from the Entner–Doudoroff pathway (EDD)^16^. Furthermore, more recent evidence suggested the presence of the phosphoketolase pathway, which has been proposed to contribute to acetyl-CoA production^35^. The genomic data predict a complete TCA cycle^15^. At least three possible pathways for 2-oxoglutarate to succinyl-Co-A/succinate conversion could be identified: 1) the traditional 2-oxoglutarate dehydrogenase complex; 2) 2-oxoglutarate:ferredoxin oxidoreductase; and 3) γ-aminobutyric acid shunt^36^. However, the exact arrangement of the TCA in the methanotroph remains to be established. Furthermore, while the functional significance of the PPi-phosphofructokinase, a key enzyme of the EMP has been well established, the role of other PPi-dependent enzymes involved in central metabolism and anaplerotic CO_2_ fixation has never been evaluated. In this study, we focus on mathematical modeling of methane and methanol utilization pathways in 20Z^R^ taking into account updated metabolic networks, optimized growth parameters (growth rate and biomass yields), methane/oxygen consumption, and as well as enzymatic and global non-targeted metabolomics data.

## Results and Discussion

### Growth optimization: impact of microminerals

It could be predicted, that the growth of the *M. alcaliphilum* 20Z^R^ will depend on availability of Cu, Ca (or La) and W. The *M. alcaliphilum* 20Z^R^ does not grow without copper, and the optimal growth is observed between 5–10 µM of Cu (II) (data not shown). As tungsten is not a standard component of methanotrophic growth media, a set of batch cultivation experiments with/without tungsten (W) was performed to examine its effects on growth and formate excretion. The growth rate of the culture did not change, however the final biomass yield increased by 7.5 ± 2.5%. Concentrations of C_1_-C_4_ organic acids in the growth medium were measured using NMR as described previously^16^. We found that formate production in cells grown in the presence of tungsten is reduced significantly, suggesting that tungsten limitation is a major factor contributing to formate excretion (Table 1). The data confirmed that NAD-linked, tungsten-dependent formate dehydrogenase is the main enzyme predicted for formate oxidation in *M. alcaliphilum* 20Z^R^. Therefore, all experiments carried-out in this work, were done with the W-supplemented growth medium. The substitution of Ca with La in the medium trace solution induced significant shifts in central metabolic pathways, which will be described elsewhere. In this study, we present data collected upon growth with calcium, i.e., with the Ca-dependent MDH, linked to cytochrome c_L_.

**Table 1:** Biomass composition and growth parameters of *Methylomicrobium alcaliphilum* 20Z. *CO_2_ production rates are below expected due to high pH of the growth medium.

Compound	%	SD	mmol/g DCW biomass	Organism source	Reference
**Amino Acids**	**43**	**2**			
Alanine			0.366	*M. alcaliphilum 20Z*	This study
Arginine			0.149	-“-	-“-
Asparagine			0.119	-“-	-“-
Aspartate			0.409	-“-	-“-
Cysteine			0.024	-“-	-“-
Glutamate			0.487	-“-	-“-
Glutamine			0.15	-“-	-“-
Histidine			0.078	-“-	-“-
Glycine			0.339	-“-	-“-
Isoleucine			0.203	-“-	-“-
Leucine			0.332	-“-	-“-
Lysine			0.182	-“-	-“-
Methionine			0.093	-“-	-“-
Phenylalanine			0.171	-“-	-“-
Proline			0.161	-“-	-“-
Serine			0.194	-“-	-“-
Threonine			0.229	-“-	-“-
Tryptophan			0.072	-“-	-“-
Tyrosine			0.111	-“-	-“-
Valine			0.278	-“-	-“-
**Ectoine**	**1.5**	**0.1**	0.106	-“-	-“-
**Lipids**	**14**	**1**			
**FAME (8.8%)**					
C14 (myristate)			0.010	*M. alcaliphilum 20Z*	-“-
C15 (pentadecylic acid)			0.002	-“-	-“-
C16 (palmitate)			0.329	-“-	-“-
C18 (stearate)			0.002	-“-	-“-
Phosphatidylserine (1%)*			0.002	-“-	^26^
Phosphatidylethanolamine (50%)*			0.086	-“-	-“-
Dipalmitoyl phosphatidate (4)*			0.007	-“-	-“-
Cardiolipin (11)*			0.019	-“-	-“-
Phosphatidylglycerol (34)*			0.059	-“-	-“-
**Sterols and bacteriohopanepolyol**	**0.02**				
tetrahymanol			0.025	*M. alcaliphilum 20Z*	^59^
aminotriol (III), 65% of BHP			0.008	-“-	^60^
3-Me-aminotriol, 31% BHP			0.003	-“-	-“-
aminotetrol and 3-Me-aminotetrol, <3% BHP			-“-	-“-	0.001
**Intracellular metabolites**	**1.5**	—			
Ribulose-5-phosphate/Ribose-5-phosphate			0.001	*M.aclaliphilum* 20Z	16
Fructose-1, 6-bisphosphate			0.001	-“-	-“-
Fructose-6-phosphate			0.003	-“-	-“-
Glucose-6-phosphate			0.002	-“-	-“-
Glyceraldehyde-3-phosphate/Dihydroxyacetone			0.003	-“-	-“-
6-Phosphogluconic acid			0.00015	-“-	-“-
2-dehydro-3-deoxy-phosphogluconate			0.000003	-“-	-“-
Phosphoglycerate			0.006	-“-	-“-
Phosphoenolpyruvate			0.005	-“-	-“-
Pyruvate			0.015	-“-	-“-
Acetyl-CoA			0.0001	-“-	-“-
Succinate			0.002	-“-	-“-
Malate			0.004	-“-	-“-
Fumarate			0.001	-“-	-“-
Citrate			0.001	-“-	-“-
Glycerate			0.001	-“-	-“-
ATP			0.005	*Methylomonas methanica*	^61^
ADP			0.002	*M. extorquens* AM1	^62^
AMP			0.001	*M. extorquens* AM1	-“-
NAD			0.002	*M. extorquens* AM1	-“-
NADH			0.002	*M. extorquens* AM1	-“-
NADP			0.001	*M. extorquens* AM1	-“-
NADPH			0.001	*M. extorquens* AM1	-“-
polyP (PPi)			0.029	*Methylomonas methanica*	^61^
**Cofactors**					
Cytochrome c			0.00036	*M. alcaliphilum 20Z*	^37^
B12			0.000000063	-“-	^63^
Ubiquinol-8			0.00022	Assumption	^62^
Protoheme			0.00022	Assumption	-“-
coenzyme-A			0.00022	Assumption	-“-
FMN			0.00022	Assumption	-“-
FMNH2			0.00022	Assumption	-“-
FAD			0.00022	Assumption	-“-
SAM			0.00022	Assumption	-“-
Glutathione			0.00022	Assumption	-“-
**Carbohydrates**	**1.38**	0.2			
Mannose			0.002	*M. alcaliphilum* 20Z	^64^
Ramnose			0.0002	-“-	-“-
Glycogen	**8.1**	1.4	0.448	-“-	This study
Sucrose	**0.9**	0.2	0.026	-“-	^65^
Ribose			0.036	-“-	^64^
Maltose			0.008	-“-	-“-
Arabinose			0.022	-“-	-“-
Galactose			0.005	-“-	-“-
**Cell wall**					
Peptidoglycan	**9.1**	-	0.053	Assumed as in *M. buryatense 5GB1*	^24^
LPS (lipid IVA and KDO)	**0.027**	-	0.002	-“-	-“-
**RNA**	**9.7**	**3.6**			This study
ATP			0.050	*M. alcaliphilum 20Z*	-“-
UTP			0.050	-“-	-“-
CTP			0.047	-“-	-“-
GTP			0.047	-“-	-“-
**DNA**	**4.0**	**0.28**			This study
dATP			0.0021	*M. alcaliphilum 20Z*	-“-
dTTP			0.0021	-“-	-“-
dCTP			0.0020	-“-	-“-
dGTP			0.0020	-“-	-“-
**Ash**	**5**			Assumed as in *M. buryatense 5GB1*	^40^
**BIOMASS (measured)**	**98.2**	**8.8**			
3-PG (balance)	1.8		0.010		
**TOTAL**	**100.0**				
**ELEMENTAL COMPOSITION**					
**C**	44.9	2.6		*M. alcaliphilum 20Z*	This study
**N**	8.1	2.4		-“-	-“-
**H**	6.8	0.1		-“-	-“-
**EXCREATED PRODUCTS**					-“-
Formate			0.7	*M. alcaliphilum 20Z*	-“-
Acetate			0.02	-“-	-“-
Lactate			0.004	-“-	-“-
EPS	**0.5–1%**		**1%**	-“-	-“-
DCW (% WCW)	25 ± 3.4				
**Growth parameters**					
**Continuous culture (5% CH4: 5%O2:N2 balance)**					
Methane Uptake (mmol g CDW^−1^ h^−1^)	11.7 ± 0.14				This study
Oxygen Uptake (mmol g CDW^−1^ h^−1^)	14.7 ± 0.9				-“-
O_2_/CH_4_ Uptake Ratio	1.25 ± 0.06				-“-
CO_2_ production (mmol g CDW^−1^ h^−1^)*	0.37 ± 0.05				-“-
Specific Growth Rate (h^−1^)	0.12 ± 0.01				-“-
**Batch culture (10% CH4:Air)**					
Methane Uptake (mmol g CDW^−1^ h^−1^)	18.6 ± 1.4				This study
Oxygen Uptake (mmol g CDW^−1^ h^−1^)	24.4 ± 2.1				-“-
O_2_:CH_4_ Uptake Ratio	1.31 ± 0.07				-“-
Specific Growth Rate (h^−1^)	0.14 ± 0.02				-“-

### Biomass composition

Overall biomass of *M. alcaliphilum* 20Z^R^ cells consists of 44.9 ± 2.6% carbon (C), 8.1 ± 2.4% nitrogen (N) and 6.8 ± 0.1 hydrogen (H). The composition of main precursors (amino acids, fatty acids, nucleic acids, carbohydrates) was measured and summarized in Table 2. The main components were estimated as follows: 43 ± 2% amino acids, 14 ± 1% lipids, 9.7 ± 3.6% RNA, 8.1 ± 1.4% glycogen, 4 ± 0.3% DNA, 1.5 ± 0.1% ectoine, and 0.9 ± 0.2% sucrose. The list of intracellular metabolites, concentrations of cytochromes, ATP and PPi, phospholipids were compiled from published literature^16,25,26,37^.

**Table 2:** Accumulation of excreted organic acids (mg g^−1^ DCW) by *M.alcaliphilum* 20Z^R^ grown in batch culture with or without sodium tungsten (0.07 mg L^−1^).

Compound	−Na_2_WO_4_	+Na_2_WO_4_
Formate	45.8 ± 1.3	2.4 ± 0.9
Acetate	2.4 ± 0.2	2.0 ± 0.1
Lactate	ND	trace
Succinate	ND	trace
Pyruvate	ND	ND
Citrate	ND	ND

### Flux balance model and growth parameters

The model consists of three compartments (extracellular space, periplasm and cytoplasm) and 396 internal enzymatic reactions (represented by 407 genes), 16 transport reactions between cell compartments, 422 metabolites and 20 exchange rates for nutrients and excreted compounds (see Supplemental Material, Table S1).

Since the energetics of methane oxidation in 20Z^R^ can be linked to aerobic or anaerobic respiration and fermentation, electron transfers were further refined by incorporating aerobic respiration pathways (from NADH/H^+^, FADH_2_, and cytochrome C_L_), anaerobic respiratory pathways, and fermentation pathways^16^. As the source of electrons is still unknown, we incorporated a pMMO-specific electron carrier (e-donor), which could be reduced via three pathways: 1) a ubiquinone (UQH_2_)/**e**-carrier to represent UQ-linked methane oxidation*;* 2) a cytochrome c_L_ (cytC_L_)/**e**-carrier to represent direct coupling between pMMO and methanol dehydrogenase; and 3) cytc_L_ and cytbc1/2**e**-carriers to represent previously proposed reverse (also known as uphill) electron transfer^18,24^. The electron transfer reactions were connected to the production of proton/ion motive force (PMF), with the following ratios: electron transfer from NADH/H^+^ to ubiquinone linked to the transfer of 4 H^+^ (or 0.4 PMF); electron transfer from ubiquinol to cytochrome bc1 (cytBc1) and to cytochrome c (cytC) linked to the transfer of 4 H^+^ (or 0.4 PMF); electron transfer from cytochrome c (cytC) or from cytochrome c_L_ (cytc_L_) to cytochrome oxidase linked to the transfer of 2 H^+^ (or 0.2 PMF). The electron transfer from ubiquinol to ubiquinol oxidases was also linked to the translocation of 4 H^+^ (or 0.4 PMF). ATP production via oxidative phosphorylation was set as the following: 1 ATP produced per 3 H^+^ translocated. The initial value of non-growth and growth-associated ATP maintenance were adapted from *Methylomicrobium buryatense* 5 G(B1)^24^.

Additional constraints were added as follows: 1) only water and CO_2_ are included as the expected excretion products; excreted formate, acetate, lactate, and exopolysaccharides were incorporated as part of the biomass equation and included in the biomass flux; 2) the methane consumption rate was set at 11.7 ± 0.14 mmol g^−1^ DCW h^−1^, (DCW, dry cell weight) based on measurements taken during continuous culture growth (at observed μ = 0.12 ± 0.01 h^−1^); and 3) the oxygen consumption rate was set at >11.7 and <20.6 mmol gDCW^−1^ h^−1^. The lowest parameter of oxygen consumption is based on the stoichiometry of methane oxidation, as 1 mol of oxygen is needed for oxidation of 1 mol methane. The upper limit represents the highest oxygen consumption rate observed during methane-limited growth (unpublished data). The oxygen consumption rate during exponential growth in continuous culture was 14.7 ± 0.9 mmol g^−1^ DCW h^−1^.

The initial metabolic model was further refined using enzymatic data (compiled in Table 3), and published ^13^C-carbon-labeling analyses, and transcriptomic data^16^. A number of central metabolic pathway reactions, including a set of reactions for the anaplerotic carbon fixation and PPi-linked reactions^29^ were validated and the following modifications were made: 1) both oxaloacetate and malate were set as key entry points for CO_2_ fixation, via pyruvate carboxykinase (PC, EC 6.4.1.1) or PP_i_-phosphoenolpyruvate carboxykinase (PEPCK, EC 4.1.1.38) and malic enzyme reactions (ME, EC 1.1.1.40), respectively; 2) interconversions between pyruvate and phosphoenolpyruvate were via pyruvate kinase (PYC, EC 2.7.1.40) and pyruvate phosphate dikinase (PPDK; EC 2.7.9.1); (3) all variants of the RuMP and the serine cycle were incorporated into the model;^24,35^ (4) all possible variants of the TCA cycle were included^15,36^.

**Table 3:** Summary of kinetic parameters for enzymes from central metabolic pathways in *M.alcaliphilum* 20Z(^R^).

Enzyme	Cofactor	Enzyme activity (or Vmax) nmol min^−1^ mg protein^−1^	Km/Ks (mM)	References
Methane monooxygenase (pMMO)	whole cells assay	120 ± 40		^64^
Methanol dehydrogenase	PMS/Ca	230 ± 10		-“-
Formate dehydrogenase	NAD^+^	132 ± 7		-“-
NADH-dehydrogenase (with 100 mM NaCl)	NADH	454 ± 50		^37^
NADH-dehydrogenase (without NaCl)	NADH	383 ± 80		-“-
ATPase	ADP	8.6 ± 0.4		-“-
Hydroxypyruvate reductase*	NADPH	41 ± 2		^66^
	NADH	26 ± 7		-“-
Serine-glyoxylate aminotransferase,		ND		^64^
Glucokinase*	ATP	217 ± 8	0.32	^67^
3-Hexulose phosphate synthase*	Ru5P	172 ± 9	0.13	^68^
Phosphoglucose isomerase		32 ± 2		-“-
6-Phoshofructokinase*	F6P/PPi	577 ± 60	0.64	^30^
	F6P/ATP	ND		-“-
	FBP/PPi	805 ± 80	0.095	-“-
Glucose-6-phosphate dehydrogenase	NAD^+^	23 ± 2		^64^
	NADP^+^	34 ± 2		-“-
6-Phosphogluconate dehydrogenase	NADP^+^	32 ± 2		-“-
	NAD^+^	12 ± 1		-“-
Fructose-1,6-bisphosphate aldolase		35 ± 2		^64^
KDPG aldolase		62 ± 3		-“-
Enolase		10 ± 1		-“-
Fructokinase			2.5	^69^
Acetate kinase*	ADP	1290 ± 31	5.6	^31^
Phosphoketolase methane methanol	TPP	0.11 ± 0.02 0.047 ± 0.009		This study
Pyruvate dehydrogenase methanemethanol	NAD^+^	120 ± 20 290 ± 44		-“-
2-Oxoglutarate dehydrogenase	NAD^+^	N.D.		-“-
Succinate semiladehyde dehydrogenase	NADP	80 ± 17.4		-“-
Glutamate decarboxylase	NAD+	7.4 ± 1.1		-“-
Pyruvate kinase*	ADP	200 ± 12*		^16^
Citrate synthase		6 ± 0.5		^64^
Isocitrate dehydrogenase	NAD^+^	ND		-“-
	NADP^+^	11 ± 1		-“-
Malate dehydrogenase*	Malate (NAD+)	1500 ± 150	0.11 (0.45)	^32^
	Oxaloacetate (NADH)	2100 ± 200	0.34 (0.025)	-“-
Malate lyase	ATP, CoA	ND		^64^
Glutamate dehydrogenase	NADH	4 ± 0.5		-“-
	NADPH	3 ± 0.2		-“-
Alanine dehydrogenase	NADPH	ND		-“-
	NADH	2 ± 0.1		-“-
Glutamate synthase	NADPH	14 ± 1		-“-
Glutamine synthetase	Mn^2+^, ATP	204 ± 10		-“-

^*^Purified enzyme.

**Figure 1:**
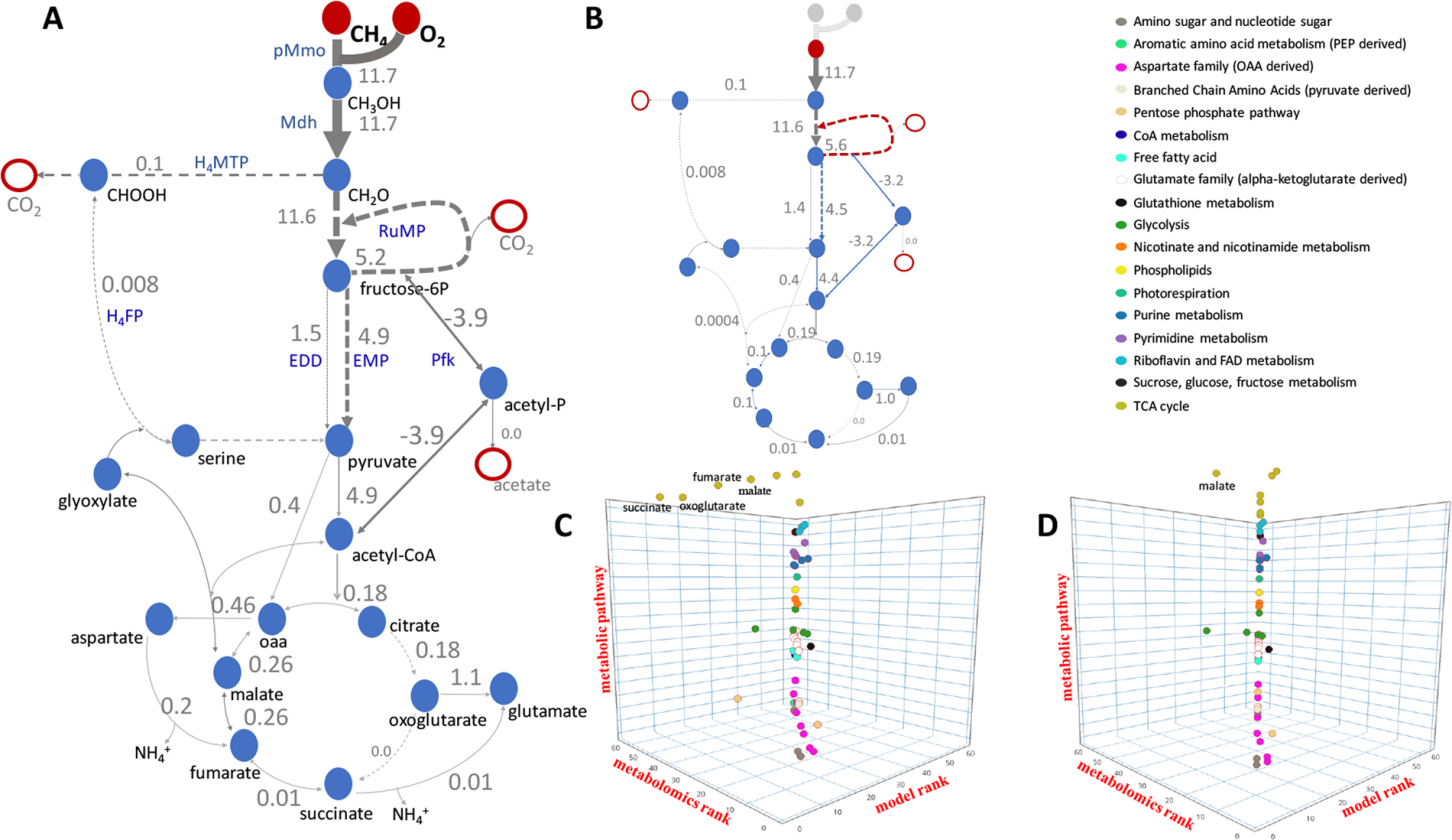
Central metabolic pathways of methane (**A**) and methanol (**B**) utilization in *Methylomicrobium alcaliphilum* 20Z^R^ and results of FBA analyses. Red circles indicate input substrates (CH_4_, O_2_, or CH_3_OH), White circles with red outlines indicate excreted compounds (CO_2_ and acetate); thickness of arrow represents predicted value of the corresponding flux via the reaction (solid arrow) or metabolic pathway (dotted arrows); (**C**,**D**) 3D plots of comparative analysis of metabolic profiling versus predicted flux ratios between the same growth conditions (axis X – model rank; axis Y – metabolomics rank; axis Z – metabolic pathway that a compound belongs to). (**C)** Comparison of results for complete TCA cycle (Spearman’s index equals to 0.9, p-value = 2.7E-24); (**D**) Comparisons of results for branched TCA cycle and reverse phosphoketolase reaction (R = 0.99, p-value = 2E-55).

An overview of the central metabolic pathways is shown in Fig. 1 (a detailed figure is provided as supplemental Figure S1). We ran a set of *in silico* flux balance analysis (FBA) experiments using the COBRA toolbox to validate the stoichiometric completeness of the proposed metabolic network at all steps of the model development. Simulations for unconstrained network supported the direct coupling (electrons for methane oxidation comes from cytC_L_) as the most optimal solution for methane oxidation, however the predicted O_2_/CH_4_ consumption was low (Table 4). As could be predicted, incorporation of PPi-reactions slightly improved biomass yield. An additional constraint on intracellular source of PPi, with biosynthesis reactions as the only source, lead to the reduction of biomass and an increase in O_2_ consumption. However, even after the modification, the predictions differ from experimental measurements (Table 4). Specifically, the predicted growth rate was higher (0.145 vs 0.12), while predicted O_2_:CH_4_ consumption rate was low (1.21 vs 1.26). Two possible explanations were explored further: 1) ATP maintenance in methanotrophic bacteria is higher than assumed^38^; and 2) ubiquinol (UQH_2_) serve as supplemental sources of electrons for methane oxidation. The ATP requirements in methane grown cells of *M. alcaliphilum 20Z*^*R*^ were measured, and the contribution of UQH2 was investigated by further constraining metabolic networks to observed oxygen consumption rate.

**Table 4:** *In silico* predictions for different metabolic arrangements of methane utilization.

Network	O_2_:CH_4_	CO_2_ production (mmol g CDW^−1^h^−1^)	growth rate (h^−1^)
Source of electrons			
Cytochrome c_L_	1.2	5.2	0.146
Ubiquinol	1.50	7.7	0.09
Initial network*	1.2	5.2	0.146
CO_2_ fixation via PEPCK	1.2	5.2	0.147
PPi-reactions (PFK, PEPCK, PPDK, PPi-ase)	1.19	5.1	0.149
Biosynthesis as a sole source of PPi (without PPiase)	1.21	5.3	0.145
Reversible PPK	1.18	5.1	0.15
Branched TCA	1.18	5.1	0.15
Fermentation mode (without ATPase)	1.02	1.5	0.06
**Final network O**_**2**_ -input constrained**	**1.26**	**5.7**	**0.136**
Experimental data	**1.26–1.31**	**-**	**0.12–0.14**

^*^Methane uptake is set to 11.7 mmol g CDW^−1^h^−1^; ** Oxygen uptake is set to 14.7 mmol g CDW^−1^h^−1^.

### ATP maintenance requirement in M. alcaliphilum 20Z^**R**^

Methane-limitation experiments were carried out, in order to estimate non-growth-associated ATP maintenance^39^. Cells of 20Z^R^ were supplied with various concentrations of methane from 0 to 2 mmol and the growth rate and methane consumption rate were measured. We found that 20Z^R^ cells require 3.6 ± 0.3 mmol CH_4_ gDCW^−1^ h^−1^ to sustain metabolic activity but not grow. Methane concentrations below this parameter led to decreases in cell density, while higher methane concentrations supported cell growth. Using the assumed ATP yield Y_*ATP, max*_ = 6 mol ATP per one mol CH_4_ consumed, the non-growth-associated ATP requirement was calculated as 21.6 mmol ATP g DCW^−1^ hr^−1^.

**Figure 2:**
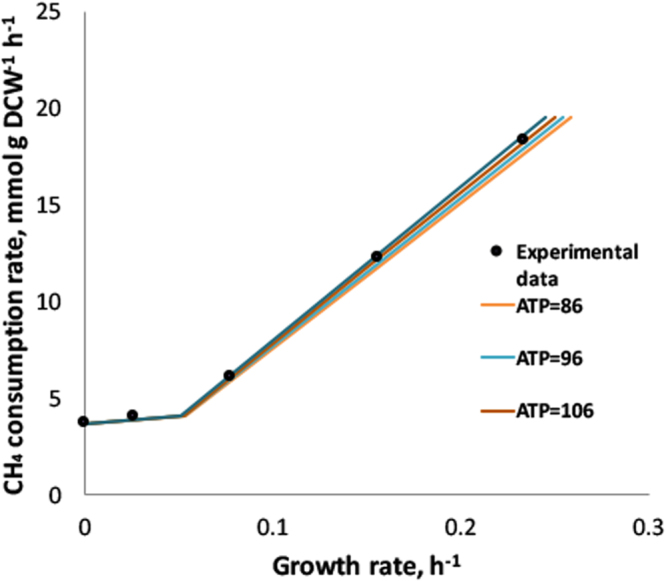
Experimentally measured (dots) and predicted growth rates for different CH_4_ consumption rates at different level of ATP maintenance. Axis X: values of growth rate (h^−1^); Axis Y: CH_4_ consumption rates (mmol g DCW^−1^ h^−1^).

Growth-associated ATP maintenance for 20Z^R^ was found by fitting the reconstructed model to the experimentally determined biomass yield. We plotted the CH_4_-uptake rate against the growth rate from continuous culture experiments (Table 1). Model simulations were carried out for various values of growth-associated ATP maintenance and the range of 90–110 mmol ATP g DCW^−1^ was found to match experimental data (Fig. 2 see Methods). While the updated maintenance coefficients improved the correlation between simulations and observed experimental data, the oxygen consumption rates were still below expected 1.26.

### Model predictions depending on simultaneous constraints of O_2_ and CH_4_ consumption

**Figure 3:**
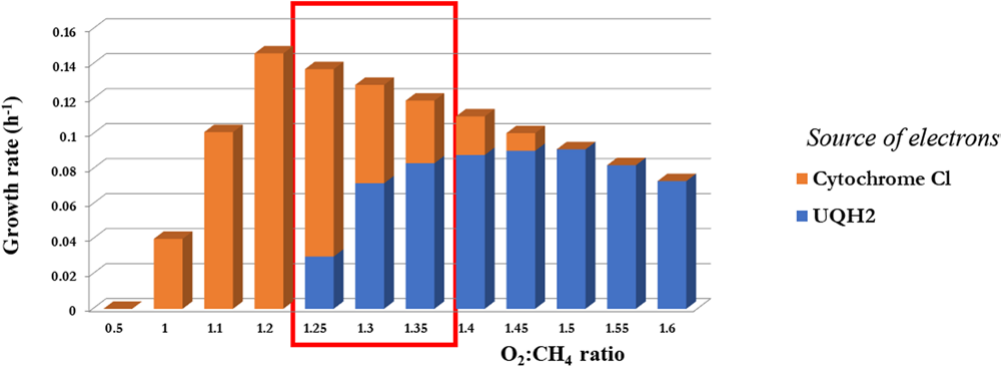
Contribution of different sources of redox for methane utilization depending on O_2_:CH_4_ ratio. The height of the colored bar reflects the biomass production or percentage of the carbon flux a specific electron donor proportionally to 100% biomass at the particular value of O_2_:CH_4_ ratio.

A set of additional *in-silico* simulations with constrained methane and oxygen consumption rates was carried out in order to investigate correlations between biomass production and O_2_/CH_4_ ratios (Fig. 3). The optimal solution for biomass production directly correlated with oxygen consumption, reaching the optimal output at 1.2–1.4—supporting direct coupling as the most compelling model for the observed O_2_:CH_4_ consumption ratio of 1.26 The contribution of electron transfer from NADH (via UQH_2_) gradually increased, ranging from 30% at a O_2_:CH_4_ consumption ratio of 1.3 to 75% at a O_2_/CH_4_ consumption ratio of 1.4, and completely replaced direct coupling at a O_2_:CH_4_ consumption ratio of 1.5. The switch to the redox arm leads to a decrease in biomass production. As the majority of the experimental data lay in the range of 1.25–1.4, optimal biomass production could not be modeled by any single mode, whether it was UQ-mediated or direct coupling. In summary, the most realistic behavior of the system could be modeled by dual simultaneous constraints on oxygen and methane consumption.

To further validate this prediction, we estimated the parameters for growth at low oxygen tension. It has been shown that upon oxygen limitation the strain switches to a fermentation mode^16^. We ran an initial unconstrained model with only a constraint on oxygen uptake (O_2_ consumption rate was set to 12 mmol gDCW^−1^ h^−1^, or O_2_/CH_4_ = 1.02). This modification resulted in significantly reduced bacterial growth (0.06 h^−1^) and an extremely elevated excretion of acetate (3.83 mmol gDCW^−1^ h^−1^). According to the *in-silico* simulations, the phosphoketolase pathway is the most compelling mode of methane fermentation, with the majority of cellular ATP produced from an acetate kinase reaction. Both the growth rate and the increase in acetate excretion have been previously observed as main outcomes of O_2_-limiting growth^16,40^. Furthermore, the kinetic properties of purified acetate kinase from *M. alcaliphilum* 20Z support the *in-silico* prediction (Table 3^31^).

### Model validation: metabolomics data

The validity of the metabolic model was further assessed by metabolomics. Metabolomic profiles were obtained for cells grown in continuous culture on methane, as well as for exponentially grown cells from batch cultures on methane and methanol. Two hundred and fifty-three compounds were identified in bacterial cell pellets. The complete list of the non-targeted metabolites is presented in Table S2 (Supplemental materials).

We conducted statistical comparisons between methane-grown cells (methane batch or optimal culture considered as control) and methanol culture condition to reveal key differences in metabolic signatures (summarized in Table S3). When compared to growth on methane, the most noticeable changes in cells grown on methanol were the increase of glycine (5.7-fold), aspartate (3.7-fold), glutamine (3.1-fold), glucose (4.8-fold), fructose (2.1-fold), and intermediates of fatty acid degradation (i.e., 3-hydroxydecanoate, 47-fold), while the intracellular levels of the TCA Krebs cycle intermediates (citrate, aconitate, malate, fumarate) and phosphosugars (fructose 6-phosphate, hexose diphosphates, sedoheptulose-7phopshate), pyruvate and acetylphosphate were reduced. Cells grown at low oxygen tension showed metabolite patterns similar to those observed previously, and had elevated levels of fatty acids [i.e., 10-heptadecenoate (17:1n7)], lactate, and succinate ^16^.

The magnitude of changes between the two groups was compared to flux changes between the same two conditions predicted by the model. A positive and statistically significant correlation between predicted fluxes and experimental metabolomics data was observed. For example, the model predicts an increased carbon flux into the pool of amino acids under methanol growth, and a slight drop of the RuMP cycle. These predictions correspond to the directions of changes for metabolites in these pathways measured by biochemical profiling. Calculations of Spearman’s index, which is proper to use in the presence of a small number of observations, between these data sets have confirmed the statistically significant correlations between model predictions and experimental measurements (Fig. 1C,D).

The comparison also highlighted a set of metabolites whose changes contradict the model predictions, including intermediates of the TCA cycle, pentose phosphate pathway, and pyruvate (Fig. 1C). Additional modifications of the model and *in-silico* simulations were carried out. Changing the phosphoketolase reaction directionality (from fructose 6-phosphate → acetylphosphate + erythrose 4-phosphate to acetylphosphate + glyceraldehyde phosphate → xylulose 5-phosphate) improved model predictions for intermediates of the pentose phosphate pathway. We also considered a complete TCA cycle for methanol and methane growth, which reduced the Spearman’s index between metabolomics profiling and flux ratios to 0.9 (p-value = 2.7E-24). Simulations with a complete TCA cycle for methanol growth and an incomplete (knockout of 2-oxogluterate dehydrogenase) TCA cycle for methane growth led to the Spearman’s index of 0.94 (p-value = 1.1E-30). However, changes in fumarate and succinate levels did not correlate with predicted flux changes, so the metabolic reactions producing or consuming those compounds were investigated further. Simulations with an unusual TCA cycle^41^—in which oxoglutarate is converted to glutamate and then to succinate via succinate-semialdehyde dehydrogenase and glutamate carboxylase—and, additionally, oxaloacetate converted to aspartate and then to fumarate via aminotransferase and aspartate lyase resulted in the Spearman’s index of 0.99 (p-value = 2E-55).

Finally, a set of enzymatic analyses were set up to validate the updated metabolic network. The activity of phosphoketolase was measured in actively grown 20Z^R^ cells demonstrating that the pathway is active upon growth on methane and methanol (Table 3); however, the highest activity of phosphoketolase is found in methane-grown cells. We also detected relatively high activity of succinate-semialdehyde dehydrogenase, supporting the hypothesis of a branched TCA cycle at the oxoglutarate node.

## Concluding Remarks

Here we demonstrated the applicability of the systems approach to analyze and improve our understanding of the methane utilization network in *M. alcaliphilum* 20Z^R^. A genome-scale metabolic model of *M. alcaliphilum* 20Z^R^ was constructed and refined using continuous cell culture parameters.

The flux balance analysis of the metabolic model quantified initial steps of methane oxidation, RuMP cycle, EMP/EDD pathways, TCA cycle; major routes for nucleotide, amino acid, and lipid biosynthesis, and fatty acid metabolism; as well as biomass growth under different conditions of the strain cultivation.

The model analysis has also identified the crucial role of an additional constraint on the O_2_ consumption rate. Due to the high plasticity of the redox reactions in the strain, the additional constraint on O_2_-input helped the model to navigate across the metabolic network and correctly reproduce experimentally observed parameters (growth rate and corresponding yields), and thus make accurate predictions.

Furthermore, we show that the integration of non-targeted metabolomics data with metabolic flux predictions can help to refine the central metabolic pathways, by highlighting nodes poorly resolved by *in-silico* reconstruction. The model simulations suggest that PPi-dependent reactions in the central metabolic pathways might contribute to energy preservation upon oxygen limitation, as well as improve carbon conversion efficiency. Global, non-targeted, metabolomic profiling combined with *in-silico* simulations uncovered a set of highly interconnected nodes at the level of acetyl-CoA, fumarate, and glutamate. Further enzymatic studies confirmed the carbon flux from acetyl-CoA to xulylose-5-phosphate via phosphoketolase, from glutamate to succinate via succinate-semialdehyde dehydrogenase and glutamate carboxylase, and from aspartate to fumarate via aspartate lyase. The model will serve to inform hypothesis-driven strain engineering strategies, targeting enhanced methane conversion rate and efficiency.

## Methods

### Metabolic modeling

To build the metabolic framework for 20Z^R^ we used the whole genome sequence of *Methylomicrobium alcaliphilum* 20Z (NC_016112.1)^15^ and the previously published metabolic reconstruction for the closely related species, *Methylomicrobium buryatense* 5 G(B1)^24^. Each reaction in these metabolic pathways was checked for mass and charge balances except reactions coupled with electron transfer system. The model was developed in a format compatible for FBA^42–44^. Moreover, to standardize reaction and metabolite IDs in our model according to BiGG Models ID Specification and Guidelines^45^, we used iAF692 model data^46^ for almost all of entities and KEGG abbreviations for enzymatic reactions, for which we could not find corresponding BiGG reactions. To link genes with reactions, the NCBI Reference Sequence annotation, NC_016112.1, was applied. To mathematically model the methane utilization network and to solve FBA optimization problems we employed GNU Linear Programming Kit (GLPK) (http://www.gnu.org/software/glpk/) solver in MATLAB using the well-known COBRA toolbox designed for flux balance model reconstruction and analyses^47^. To visualize metabolic networks and obtained fluxes we employed the Escher web-tool (Figure S1^48^).

### Strain and growth media

*M. alcaliphilum* 20Z^R^ cells were grown using modified P media (g/L): KNO_3_, 1; MgSO_4_ x 7H_2_O, 0.2; CaCl_2_ x 2H_2_O, 0.02; NaCl, 30; trace solution, 1 ml/L of trace solution^9^; and supplemented with 20 ml/L of phosphate solution (5.44 g KH_2_PO_4_; 5.68 g Na_2_HPO_4_) and 20 ml/L of 1 M carbonate buffer. The following methane mixtures were used for bioreactor studies: (i) 5% CH4: 3.5% O_2_ to represent oxygen-limiting conditions; (ii) 5% CH_4_: 5%O_2_, to represent optimal growth; and (iii) 2.5%CH_4_:10% O_2_ to represent methane-limiting conditions. Methane mixtures were directly purged into bioreactor culture at 0.1 sL h^−1^ rate. In batch cultures, methane (99.9%, Airgas) was injected into vials to represent 10–20% of the headspace. For methanol growth cultures were supplemented with 0.5% methanol.

### Cultivation

Culturing was carried out in either closed vials (50 ml cultures in 250 ml vials, or 200 ml cultures in 1 L bottles) with shaking at 200 r.p.m. or bioreactor cultures (fed-batch or turbidostat). Two types of bioreactors were used: 1) a DASbox mini bioreactor (0.5 L working volume; 200 ml culture) with two individual bioreactor units, each having automatic temperature, pH, and DO controls, a sample port for measuring OD, and a coupling to a BlueSens sensor system for simultaneous measuring off-gases (CH_4_, O_2_, and CO_2_); or 2) a 2.7 L bench top BioFlo 110 modular bioreactor (New Brunswick Scientific, Edison, NJ, USA).

Batch cultures were grown in 125 ml, 250 ml, or 1.2 L bottles. The headspace:medium was set at 4:1 ratio. CH_4_, O_2_, and CO_2_ composition of the headspace were determined using an SRI GC system. Control set of samples include only ininoculated media. The methane and oxygen consumption and CO_2_ production rates were calculated by estimating decline(or increase) of the corresponding compounds over time. The data were analyzed to assess yield (Y), growth rate, and O_2_/substrate ratios. In addition, samples (15 ml) were taken for metabolomics.

### ATP maintenance experiments

To estimate growth-dependent and non-growth associated ATP maintenance cells of 20Z^R^ were pre-grown to OD = 0.36 ± 0.05 and transferred to new vials supplemented with various amounts of methane added to headspace (0, 0.02, 0.04, 0.08, 0.1, 0.2, 0.4, 0.8, 1.6 mmol). Each experiment was represented by 3–6 biological replicates. Vials with cultures were placed back onto shaking platform. OD and CH_4_, O_2_ and CO_2_ composition were measured every 30 min – 1 h over 12 hours. The growth rates of methanotrophic bacteria correlated with the initial concentrations of substrate added. No growth was observed in cultures supplemented with methane below 3.6 mmol g^−1^ CDW h^−1^.

Methane utilization for maintenance and growth is mathematically expressed as:

${q}_{C{H}_{4}}={m}_{C{H}_{4}}+\frac{\mu }{{Y}_{max}}$, where ${m}_{C{H}_{4}}$ is the methane requirement for non-growth–associated maintenance activities, *Y*_*max*_ is the maximum biomass yield including growth–associated maintenance, and μ is the growth rate^39^. These ATP requirements were determined from a plot of methane uptake versus optimal computed growth rate, given the experimentally determined values for ${m}_{C{H}_{4}}$ and *Y*_*max*_. ${m}_{C{H}_{4}}$ has been observed to have value equals to 3.6 mmol CH_4_ g DCW^−1^ h^−1^, while *Y*_*max*_ has a value 0.6 gDCW per g of CH_4_. Using the assumed ATP yield Y_ATP, max_ = 6 mol ATP per one mol CH_4_ consumed and the y-intercept (3.6 mmol CH_4_ g DCW^−1^ hr^−1^), the non-growth-associated ATP requirement was calculated as 21.6 mmol ATP g DCW^−1^ hr^−1^. To estimate growth-associated ATP maintenance the model was simulated by varying only the CH_4_ consumption rate upon different values of growth-associated ATP maintenance as a stoichiometric coefficient in the biomass equation (‘BOF’ in Supplemental Material, Table S1). Figure 2B demonstrates agreement between the equation and the flux balance approach and the corresponding growth-associated ATP requirement is the range of 90–110 mmol ATP g DCW^−1^.

### Dry cell weight measurement

Cultures (150 ml) from bioreactors were centrifuged to collect the biomass. After careful removal of the liquid phase, tubes of known weight with biomass were weighed (to obtain wet-cell biomass weight), lyophilized overnight using a Labconco freeze-dry system and weighed again. The observed DCW parameters were as follows: 1 L of cell culture with OD = 1 corresponds to 0.336 ± 0.025 g DCW).

### Biomass composition

Samples of lyophylized biomass were submitted to AminoAcids (aminoacids.com) for complete amino acid analys, and to Matrix Genetics (Seattle, WA, http://matrixgenetics.com) for FAME derivatization and GC-MS analysis. The intracellular concentration of glycogen was determined using anthron reagent^49^; ectoine concentration was determined using an HPLC assay^50^. For glycogen measuremnts 20z^R^ biomass (dry) was lysed by incubation of with potassium hydroxide (10% KOH) at 95 °C for 1 h. After neutralization and centrifugation (14000 rpm @ 15 min, 4 °C), ethanol was added to the supernatants (two volumes) and samples were refrigerated overnight. The precipitated glycogen was resuspended in 1 ml of DI H_2_O and centrifuged again (14000 rpm, 15 min, 4 °C). 1 ml of 0.2% (w/v) anthrone reagent (95% sulfuric acid) was added to 0.2 ml resuspended glycogen and incubated for 15 minutes, after which the sample was measured using a Janeway 632OD spectrophotometer and 620 nm. The concentrations of glycogen were estimated by comparing to glycogen standards prepared in the same manner as cell samples. Extracellular polysaccharides were measured in samples of broth using a sulfuric acid assay ^51^. Extracellular concentrations of formate, acetate, and lactate were measured as previously described ^16^.

### Non-targeted metabolite profiling

Metabolomic analysis of cells and spent supernatant from cultures of the *M. alcaliphilum* 20Z^R^ was performed using Metabolon’s untargeted global biochemical profiling platform [http://www.metabolon.com/technology.aspx] and according to the published protocol^52^. The data represent accurate relative quantification only for individual compounds across all the samples which contain it. The original scale data represent raw ion counts for the integrated peaks specifically representing each compound. The scaled data is generated by simply dividing each value for a compound by the median (among detected samples), then imputing any null values with the minimum detected value. Thus, the scaled data are centered around 1.0 (median) for all compounds, but the variation among the samples is not affected^53,54^. Statistical analyses were performed on natural log-transformed data.

### Statistical comparison between predicted flux changes and biochemical profiling

Statistical comparison between methane-grown cells (methane batch) and methanol culture condition was initially conducted to reveal key differences in metabolic signatures. The magnitudes of changes between two groups were compared to predicted flux changes between the same two conditions. An enzymatic reaction with the highest value of flux for biosynthesis of corresponding metabolite was used to calculate flux ratio between two growth conditions. In order to estimate Spearman’s correlation index, predicted flux ratios and metabolic signatures were ranked according to the method^55^. 3D plots reflected metabolomics and model ranks taking into account metabolic pathways for measured substances were generated with assistance of Plotly tool (Plotly Technologies Inc. Collaborative data science. Montréal, QC, 2015; https://plot.ly).

### Isolation of soluble protein fraction

Cell lysates were generated from biomass cultivated under above described conditions. Cells were pelleted via centrifugation for 1 min at 13,000 *g* and culture supernatants were discarded. Cell pellets were quenched in liquid nitrogen, thawed and solubilized on ice in 1 mL of lysis buffer (50 mM MOPS, pH 7.4, 50 mM NaCl, supplemented with 1X cOmplete Protease Inhibitor Cocktail solution (Roche Diagnostics Corporation, Indianapolis, IN)). The cells were then sonicated on ice at 4 °C, at 90% power setting for 30 seconds × 3 cycles, with a one minute cool-down period between sonication cycles using a Braun-Sonic-L ultrasonicator. Lysates were cleared via centrifugation at 16,000 × *g* at 4 °C for 5 minutes, and the supernatants were isolated for use in subsequent enzymatic analysis.

### Enzyme activity determination

Cell lysates were generated from biomass cultivated under above described conditions. Cells were pelleted via centrifugation for 1 min at 13,000 g and culture supernatants were discarded. Cell pellets were quenched in liquid nitrogen, thawed and solubilized on ice in 1 mL of lysis buffer (50 mM MOPS, pH 7.4, 50 mM NaCl, supplemented with 1X cOmplete Protease Inhibitor Cocktail solution (Roche Diagnostics Corporation, Indianapolis, IN)). The cells were then sonicated on ice at 4 °C, at 90% power setting for 30 seconds × 3 cycles, with a one minute cool-down period between sonication cycles using a Braun-Sonic-L ultrasonicator. Lysates were cleared via centrifugation at 16,000 g at 4 °C for 5 minutes, and the supernatants were isolated for use in subsequent enzymatic analysis.

Enzymatic assays for pyruvate dehydrogenase (PDH), alpha ketoglutarate dehydrogenase (AKGDH), succinate semialdehyde dehydrogenase (SSADH), and glutamate carboxylase (GC) were conducted using methodology adapted from^56^. Briefly, enzymatic activity was quantitated via continuous spectrophotometric rate determination at 340 nm following enzyme incubation with substrate, Coenzyme A, and beta-nicotinamide adenine dinucleotide (or oxidized nicotinamide adenine dinucleotide for SSADH) at 30 °C. Final assay reaction concentrations for PDH, AKGDH, and GC were as follows: 50.8 mM MOPS, 0.2 mM MgCl2, 0.01 mM CaCl2, 0.3 mM cocarboxylase (thiamine pyrophosphate), 0.12 mM coenzyme A, 2.0 mM beta-nicotinamide adenine dinucleotide, 2.6mM L-cysteine, 5 mM substrate, and 25ug total protein. Final reaction concentrations for SSADH were as follows: 87 mM potassium pyrophosphate, 3 mM 2-mercaptoethanol, 1.3 mM ß-nicotinamide adenine dinucleotide phosphate, 5.0 mM succinic semialdehyde, 0.83% (v/v) glycerol, 2.5 mM potassium phosphate, and 25ug total protein.

Phosphoketolase activity was measured using a hydroxymate assay that detects acetyl phosphate^35,57,58^. Briefly, 100 μL reactions in reaction buffer [30 mM potassium phosphate (pH 6.5), L- cysteine hydrochloride (2 mM), sodium fluoride (20 mM), sodium iodoacetate (10 mM), thiamine pyrophosphate (1 mM), and fructose 6-phosphate (30 mM) (Sigma)] were initiated by the addition of 25 μg total protein. Reactions were incubated at 37 °C for 1 h. After incubation, 50 μL of 2 M hydroxylamine hydro- chloride (pH 7.0) was added to the reaction and incubated at room temperature for 10 min. Reactions were then terminated by adding 300 μL of a 1:1 mixture of 2.5% ferric chloride in 2 M hydrochloric acid and 10% trichloroacetic acid. A color change due to ferric-hydroxamate formation was measured at A505nm, and the concentration of acetyl-P formed during the initial reaction was calculated by regression analysis compared to known standards.

All absorbance measurements were conducted using a FLUOstar Omega microplate reader (BMG Labtech) in 96-well, round-bottom plates (Corning Costar), 300-μL reaction volumes.

### Availability of data and material

Developed genome-scale model (xls) is available on the web-site: http://sci.sdsu.edu/kalyuzhlab/.

## Electronic supplementary material

Table S1

Table S3

Supplementary material
