# Author Correction: Methane utilization in *Methylomicrobium alcaliphilum* 20Z^R^: a systems approach

**DOI:** 10.1038/s41598-018-23088-w

**Published:** 2018-03-14

**Authors:** Ilya R. Akberdin, Merlin Thompson, Richard Hamilton, Nalini Desai, Danny Alexander, Calvin A. Henard, Michael T. Guarnieri, Marina G. Kalyuzhnaya

**Affiliations:** 1 Biology Department and Viral Information Institute, San Diego State University, San Diego, USA; 2 Institute of Cytology and Genetics and Novosibirsk State University, Novosibirsk, Russia; 3 Metabolon, Inc. 617 Davis Drive, Suite 400, Durham, NC 27713 USA; 4 National Bioenergy Center, National Renewable Energy Laboratory, 15013 Denver West Parkway, MS 3323, Golden, CO 80401 United States

Correction to: *Scientific Reports* 10.1038/s41598-018-20574-z, published online 06 Fabruary 2017

The Acknowledgements section in this Article is incomplete.

“The study was financially supported by DOE under FOA DE-FOA-0001085 and by NSF-CBET award 1605031.”

should read:

“The study was financially supported by DOE under FOA DE-FOA-0001085 and by NSF-CBET award 1605031. The views and opinions of the authors expressed herein do not necessarily state or reflect those of the United States Government or any agency thereof. Neither the United States Government nor any agency thereof, nor any of their employees, makes any warranty, expressed or implied, or assumes any legal liability or responsibility for the accuracy, completeness, or usefulness of any information, apparatus, product, or process disclosed, or represents that its use would not infringe privately owned rights.”

