# Supplementary material for: Methane utilization in Methylomicrobium alcaliphilum 20ZR: a systems approach
Source: Sci Rep. 2018 Feb 6;8:2512. doi: 10.1038/s41598-018-20574-z (PMC5802761; doi:10.1038/s41598-018-20574-z)
Supplement: Supplementary file 3 — Supplementary material [file 41598_2018_20574_MOESM3_ESM.pdf]

Methane utilization in *Methylomicrobium alcaliphilum* 20Z^{R}: a systems approach

Ilya R. Akberdin, Merlin Thompson, Richard Hamilton, Nalini Desai, Danny Alexander, Calvin A. Henard, Michael T. Guarnieri, and Marina G. Kalyuzhnaya.

Supplementary Material

Table S3. Summary of non-targeted metabolomics profiling of *Methylomicrobium alcaliphilum* 20ZR cells grown at various conditions.

	RATIO	Meet Criteria	Up | Down
RELATIVE TO continuous culture (methane growth)	METHANE LOW O_2	68	59 | 9
	METHANE:ETHANE	93	80 | 13
	METHANOL	92	73 | 19
RELATIVE TO batch culture (methane)	CONTINUOUS CULTURE	42	5 | 37
	METHANE LOW O_2	26	6 | 20
	METHANE: ETHANE	22	12 | 10
	METHANOL	50	24 | 26

Figure S1. Metabolic network of methane and methanol utilization in *Methylomicrobium alcaliphilum* 20Z

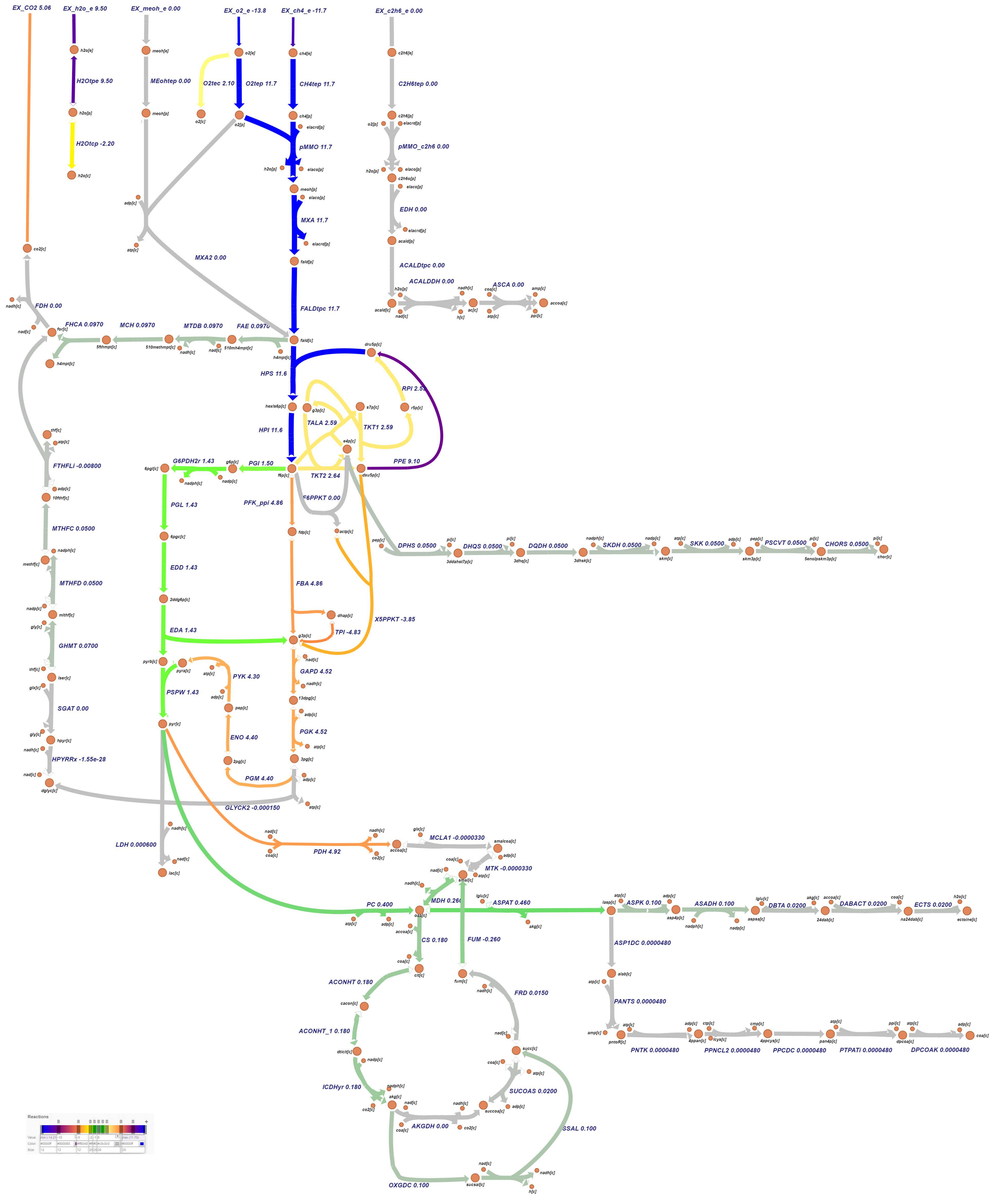

EX_CO2 5.06
EX_h2o_e 9.50
EX_meoh_e 0.00
EX_o2_e -13.8
EX_ch4_e -11.7
EX_c2h6_e 0.00
H2Otpe 9.50
MEohtep 0.00
O2tec 2.10
O2tep 11.7
CH4tep 11.7
C2H6tep 0.00
H2Otcp -2.20
pMMO 11.7
pMMO_c2h6 0.00
MXA 11.7
EDH 0.00
MXA2 0.00
ACALDtpc 0.00
ACALDDH 0.00
ASCA 0.00
FDH 0.00
FALDtpc 11.7
FHCA 0.0970
MCH 0.0970
MTDB 0.0970
FAE 0.0970
HPS 11.6
RPI 2.51
TALA 2.59
TKT1 2.59
HPI 11.6
FTHFLi -0.00800
G6PDH2r 1.43
PGI 1.50
TKT2 2.64
PPE 9.10
PFK_ppi 4.86
F6PPKT 0.00
PGL 1.43
MTHFC 0.0500
DPHS 0.0500
DHQS 0.0500
DQDH 0.0500
SKDH 0.0500
SKK 0.0500
PSCVT 0.0500
CHORS 0.0500
EDD 1.43
MTHFD 0.0500
FBA 4.86
X5PPKT -3.85
EDA 1.43
TPI -4.83
GHMT 0.0700
GAPD 4.52
PYK 4.30
PSPW 1.43
SGAT 0.00
ENO 4.40
PGK 4.52
HPYRRx -1.55e-28
PGM 4.40
GLYCK2 -0.000150
LDH 0.000600
MCLA1 -0.0000330
PDH 4.92
MTK -0.0000330
MDH 0.260
PC 0.400
ASPAT 0.460
ASPK 0.100
ASADH 0.100
DBTA 0.0200
DABACT 0.0200
ECTS 0.0200
CS 0.180
FUM -0.260
ASP1DC 0.0000480
ACONHT 0.180
FRD 0.0150
PANTS 0.0000480
ACONHT_1 0.180
PNTK 0.0000480
PPNCL2 0.0000480
PPCDC 0.0000480
PTPATI 0.0000480
DPCOAK 0.0000480
ICDHyr 0.180
SUCOAS 0.0200
AKGDH 0.00
SSAL 0.100
OXGDC 0.100
Reactions
